# Long-lasting insecticidal nets retain bio-efficacy after 5 years of storage: implications for malaria control programmes

**DOI:** 10.1186/s12936-020-03183-y

**Published:** 2020-03-14

**Authors:** Jeremiah J. Musa, Sarah J. Moore, Jason Moore, Emmanuel Mbuba, Edgar Mbeyela, Dickson Kobe, Johnson K. Swai, Olukayode G. Odufuwa

**Affiliations:** 1 Environmental Health and Ecological Science Department, Ifakara Health Institute, P.O. Box 74, Bagamoyo, Tanzania; 2 Department of Life Science and Bio-Engineering, The Nelson Mandela African Institution of Science and Technology, P. O. BOX 447, Arusha, Tanzania; 3 University of Basel, St. Petersplatz 1, 4002 Basel, Switzerland; 4 Swiss Tropical and Public Health Institute, Socinstrasse. 57, 4002 Basel 4, Switzerland

**Keywords:** Long storage nets, Long lasting insecticidal nets, LLIN, ITN, Malaria, Tanzania

## Abstract

**Background:**

Long-lasting insecticidal nets (LLINs) are the most sustainable and effective malaria control tool currently available. Global targets are for 80% of the population living in malaria endemic areas to have access to (own) and use a LLIN. However, current access to LLINs in endemic areas is 56% due to system inefficiencies and budget limitations. Thus, cost-effective approaches to maximize access to effective LLINs in endemic areas are required. This study evaluated whether LLINs that had been stored for 5 years under manufacturer’s recommended conditions may be optimally effective against *Anopheles* mosquitoes, to inform malaria control programmes and governments on the periods over which LLINs may be stored between distributions, in an effort to maximize use of available LLINs.

**Methods:**

Standard World Health Organization (WHO) bioassays (cone and tunnel test) were used to evaluate the bio-efficacy and wash resistance of Olyset^®^ and DawaPlus^®^ 2.0 (rebranded Tsara^®^ Soft) LLINs after 5 years of storage at 25 °C to 33.4 °C and 40% to 100% relative humidity. In addition, a small scale Ifakara Ambient Chamber test (I-ACT) was conducted to compare the bio-efficacy of one long stored LLINs to one new LLIN of the same brand, washed or unwashed. LLINs were evaluated using laboratory reared fully susceptible *Anopheles gambiae* sensu stricto (s.s.) (Ifakara strain) and pyrethroid resistant *Anopheles arabiensis* (Kingani strain).

**Results:**

After 5 years of storage, both unwashed and washed, Olyset^®^ and DawaPlus^®^ 2.0 (Tsara^®^ Soft) LLINs passed WHO bio-efficacy criteria on knockdown (KD60) ≥ 95%, 24-h mortality ≥ 80% and ≥ 90% blood-feeding inhibition in WHO assays against susceptible *An. gambiae* s.s. DawaPlus^®^ 2.0 LLINs also passed combined WHO bioassay criteria against resistant *An. arabiensis.* Confirmatory I-ACT tests using whole nets demonstrated that long-stored LLINs showed higher efficacy than new LLINs on both feeding inhibition and mortality endpoints against resistant strains.

**Conclusions:**

Even after long-term storage of around 5 years, both Olyset^®^ and DawaPlus^®^ 2.0 LLINs remain efficacious against susceptible *Anopheles* mosquitoes at optimal storage range of 25 °C to 33.4 °C for temperature and 40% to 100% relative humidity measured by standard WHO methods. DawaPlus^®^ 2.0 (Tsara^®^ Soft) remained efficacious against resistant strain.

## Background

Long-lasting insecticidal nets (LLINs) remain the most sustainable and effective malaria control tool available in endemic countries [1], despite insecticide resistance [2]. Approximately 663 million cases of malaria have been prevented by LLINs since the year 2000, representing 68% of the total cases averted by all interventions used for malaria control [3]. In 2007, the mass distribution of LLINs was recommended by the World Health Organization (WHO) as the core element of the global malaria strategy for malaria vector control in endemic areas [4, 5]. Between 2008 and 2016, more than 1 billion LLINs were distributed in Africa through mass campaigns [6]. The wide scale-up of LLIN distribution has led to a significant reduction of malaria morbidity and mortality [7]. Although, before a brand of LLIN can be listed as a potential product for mass campaign by the WHO, it must have undergone rigorous laboratory and field testing [8]. Currently, there are 20 brands of LLINs that are prequalified by the WHO for use in national distribution campaigns [9]. These LLINs are expected to retain their insecticidal activity by killing mosquitoes and preventing mosquito bites to confer both personal and community protection from malaria for at least 3 years (20 washes is used as a proxy for 3 years of use) [8].

The public health benefit of LLINs is attained through sustained high net access at the community level, which is referred to as universal coverage [10]. Currently, the global recommended target for population access to LLINs is > 80%, and is referred to as the minimum operational effectiveness coverage level that will translate to the community effect of LLINs [11]. Operationally, this is defined as one net used per two people (de facto) in the population [12, 13].

The governments of malaria endemic countries and international donors such as Global Fund, U.S. President’s Malaria Initiative (PMI) as well as non-governmental organisations (NGOs) have been providing funds for procurement of LLINs and related logistics to ensure high access to LLINs through multiple channels [14, 15]. Nevertheless, access to LLINs was 56% in endemic regions [16]. Even shortly after mass distribution campaigns of LLINs, population access rarely exceeds 80% [6, 12, 17]. In Tanzania, access to LLINs was 50% [16] and trends in malaria burden declines in years after mass campaigns and then increases in the following years as LLINs wear out. Insufficient access to LLINs is mainly due to long intervals between net distribution campaigns, population growth, inadequate funds and budget limitations on malaria control programmes [5, 16–18]. Increasing access to LLINs through cost-effective solutions remains a critical concern and a number of strategies are being explored for “keep-up campaigns” to retain high LLIN access [13].

It is known that the correct storage of LLINs is important to retain their bio-efficacy, before and during mass distribution campaigns. Exposure of LLINs to direct sunlight [19] and storage at high temperature degrade the pyrethroid insecticides used on LLINs [19, 20]. Even though guidance on the correct storage conditions for LLINs before and during distributions is available [21], there is limited information on the maximum storage period for LLINs before they are no longer bio-efficacious. Therefore, this study evaluated the bio-efficacy and wash resistance of Olyset^®^ and DawaPlus^®^ 2.0 (currently rebranded Tsara^®^ soft) LLINs that have been stored for more than 5 years (long storage LLINs) under optimal conditions of 25 °C to 33 °C and 40% to 100% relative humidity (RH).

## Methods

### Study design

Two brands of LLINs: Olyset^®^ and DawaPlus^®^ 2.0 (Tsara^®^ soft) that were stored for more than 5 years under recommended conditions were evaluated. The study was conducted in two stages. First, through a randomized double blinded, bio-efficacy evaluation of LLINs using standard WHO assays [8]. This was followed by partially randomized double blinded semi-field tests to compare the bio-efficacy of long stored LLINs against the new LLINs of the same brand using the Ifakara Ambient Chamber tests (I-ACT) [22]. Untreated Safi Net was used as a negative control in all tests to monitor the quality of the experiment.

### Test facility

The experiments were performed at the Vector Control Product Testing Unit (VCPTU) of the Ifakara Health Institute located in Bagamoyo, Tanzania. (http://ihi.or.tz/static/media/Vector-Control-Product-Testing.e31c173f.pdf).

### Test nets

Olyset^®^ is a high-density mono-filament polyethylene LLIN, incorporated with 20 g/kg (± 3 g/kg), 2% w/w of permethrin (corresponding to 1000 mg/m^2^). Olyset^®^ is manufactured by A to Z Textile Mills Ltd, Arusha, Tanzania. Tsara soft formally known as DawaPlus^®^ 2.0 is manufactured by NRS Moon Netting FZE [9]. Tsara^®^ soft is a deltamethrin-coated LLIN. The target dose of deltamethrin coated on a knitted multi-filament polyester fiber is 2.0 g/kg ± 25% with 100-denier yarn (corresponding to 80 mg/m^2^ deltamethrin). Untreated Safi Net is made of polyester fibres, manufactured by A to Z Textile Mills Ltd, Arusha, Tanzania. All nets were double sized and coded by an independent IHI staff, to allow blinding of investigators and participants. All test nets have WHO-PQ listing [9].

### Net storage conditions

Olyset^®^ and DawaPlus^®^ 2.0 LLINs were stored for approximately 5 years in the Ifakara Health Institute (IHI-Bagamoyo) storage facility and are denoted in this study as long storage (LS) nets. All nets were received directly under similar conditions from the manufacturer and were manufactured shortly before shipping for the purpose of product evaluation. LS Olyset^®^ LLINs with batch number L2605 were manufactured in May, 2013 and logged into the IHI-Bagamoyo storage facility on 4th June, 2013. LS DawaPlus^®^ 2.0 LLINs were regular production manufactured in November, 2013 and were logged into the IHI-Bagamoyo storage facility on 4th December, 2013.

The new Olyset^®^ LLINs were manufactured in 2017 with Batch number 7X15BZS, and were logged into the IHI-Bagamoyo storage facility on 22nd December, 2018. The new DawaPlus^®^ 2.0 LLINs were test series manufactured in March, 2018, with batch number 18SPL005, were shipped from the manufacturer on 15th May, 2018 and logged into the IHI-Bagamoyo storage facility on 1st June, 2018. All nets were stored and maintained at an average temperature of 29 °C [25 °C to 33.4 °C] and 40% to 100% relative humidity (RH) in the IHI-Bagamoyo storage facility. Temperature was recorded and logged each afternoon at 14:00 h which coincides with peak temperatures.

### IHI-Bagamoyo LLINs storage facility

**Fig. 1:**
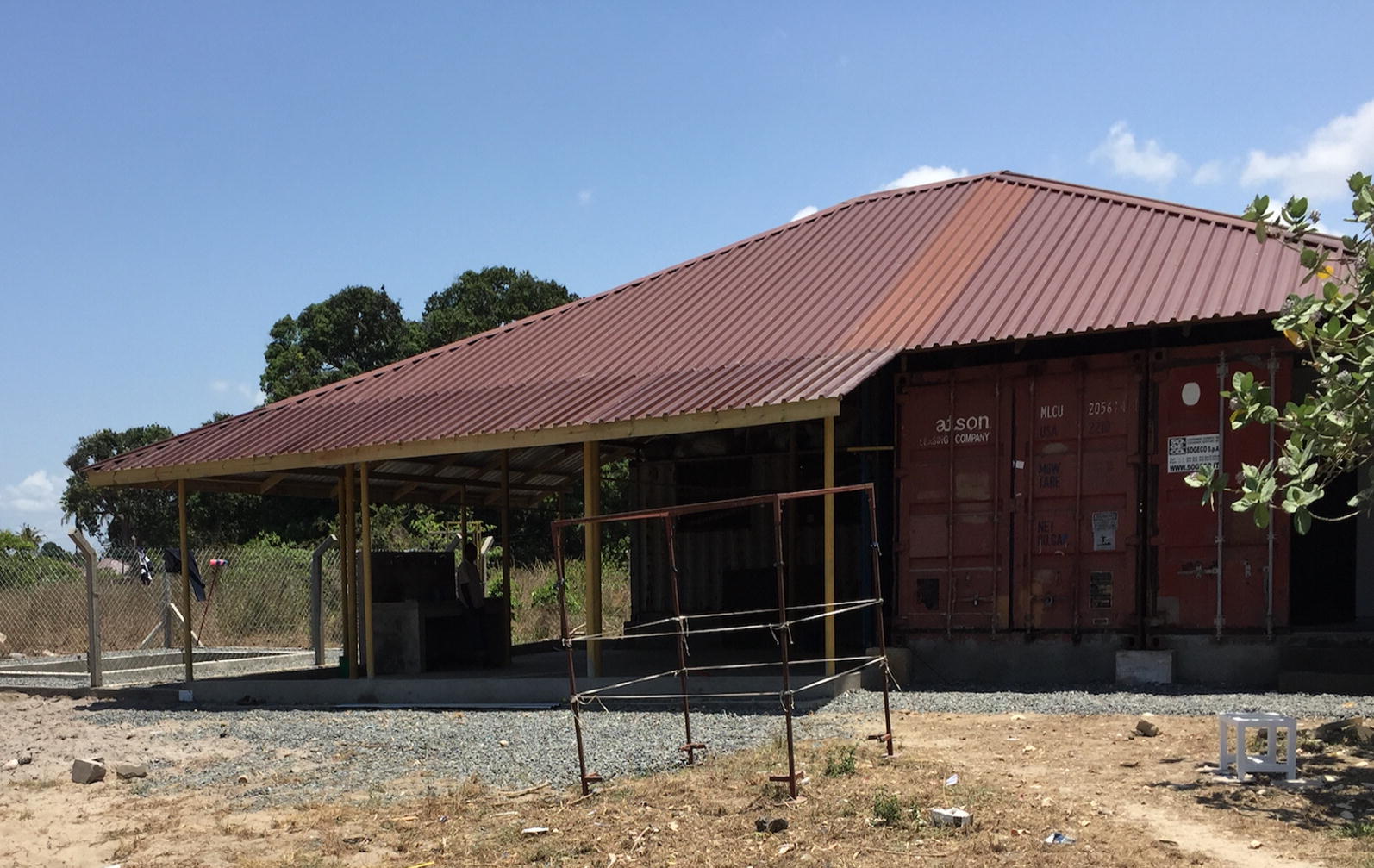
The Bagamoyo IHI LLIN storage facility

The LLIN storage used in this study was a shipping container that uses only passive cooling for the majority of the year. The container is raised above the ground and is situated under a second shade roof to reduce the radiant transfer of heat. It is installed with aluminium heat reflecting foil between the rafter and iron sheet of the storage for efficient cooling, also equipped with ventilation gaps (similar to the eaves of African houses) to allow air movement through the store. Electric ceiling fans are used only at the hottest times of the year irrespective of the temperature (Fig. 1).

The experiments were conducted from 25th January 2019 to July 2019. Olyset^®^ LLINs had been stored for 5 years and 2 months while DawaPlus^®^ 2.0 LLINs had been stored for 4 years and 8 months at the time of WHO cone assays and tunnel testing. Olyset^®^ LLINs had been stored for 5 years and 8 months while DawaPlus^®^ 2.0 LLINs had been stored for 5 years and 2 months at the time of I-ACT testing.

### LLINs preparation and washing procedures for WHO assays

Eight LLINs (four nets of each brand) were randomly selected from the same product batch. LLINs were coded, cut into pieces (25 cm × 25 cm) and washed one, three, five, ten, 15 and 20 times following WHO standard procedures for laboratory testing (phase I) [8] The washing interval of 1 day was used based on the reported regeneration time for both products [23].

### LLINs preparation and washing procedures for I-ACT assays

Eight LLINs (two old and two new DawaPlus^®^ 2.0; and two old and two new Olyset^®^) and two untreated nets were randomly selected from their product batches and coded. Two LLINs of each brand were washed 20 times as per WHO small-scale field trials (field wash) washing procedures, used as a standard procedure to simulate aging of nets under user conditions [8], while the other two were unwashed. All washed, unwashed and un-treated nets were deliberately holed (4 cm by 4 cm) six times, with one hole on each width and two holes on each length side, 75 cm from the top of the net as per WHO procedures [8].

### Test systems

Mosquitoes used for the evaluations were *Anopheles gambiae* sensu stricto (s.s.) (Ifakara strain) fully susceptible to all classes of insecticides and *Anopheles arabiensis* (Kingani strain) strongly resistant to all pyrethroids including, deltamethrin and permethrin (< 20% mortality with WHO discriminating doses, through metabolic P450 mechanism). In the WHO cone bioassays, nulliparous 3–5 days old female sugar-fed mosquitoes were used while in the tunnel test and I-ACT, nulliparous 5–8 days old female mosquitoes’ sugar-starved for 8 h were used. The VCPTU mosquito colonies are maintained at 27 °C ± 5 °C and 40% to 100% relative humidity with access to 10% sucrose ad libitum supplemented by membrane feeding using cow blood for the purposes of egg production following MR4 guidelines [24].

### WHO assays

Cone bioassays were conducted on unwashed and “field-washed” long storage LLINs, with four LLINs of each brand tested per condition. Five mosquitoes were exposed for 3 min per cone on each net replicate. Long storage LLINs that failed to meet WHO cone bioassay threshold criteria (Table 1), were subjected to WHO tunnel test as per WHO guideline [8].

**Table 1:** The summary of experimental design on WHO and I-ACT bioassays

Particular	WHO cone test	WHO tunnel test	Ifakara Ambient Chamber test (I-ACT)
Mosquitoes exposed	80 per net	100 per net piece	60 (30 per strain) per net
Exposure time	3 min	12 h	9 h
Mosquito holding conditions	27 °C ± 5 °C 40–100% RH	27 °C ± 5 °C 40–100% RH	27 °C ± 5 °C 40–100% RH
Mosquito status	3–5 day old female, sugar-fed, nulliparous	5–8 day old female, sugar-starved, nulliparous	5–8 day old female, sugar-starved, nulliparous
Bait	None	Rabbit	Human
Outcome measures	% KD60 (uncoordnated movement or unable to move or body impairement or rapid fall after take off by the mosquito after one hour (60mins)) % 24-h mortality	% Feeding inhibition % 24-h mortality	% Feeding inhibition % 24-h mortality
WHO efficacy criteria	≥ 95% KD60 ≥ 80% 24-h mortality	≥ 90% feeding inhibition ≥ 80% 24-h mortality	≥ 90% feeding inhibition ≥ 80% 24-h mortality
Test validity on negative control	≤ 10% mortality	≥ 50% feeding success ≤ 10% mortality	≥ 50% feeding success ≤ 10% mortality
Analysis	Descriptive analysis	Descriptive analysis	Descriptive analysis and binary logistic regression

### I-ACT assays

**Fig. 2:**
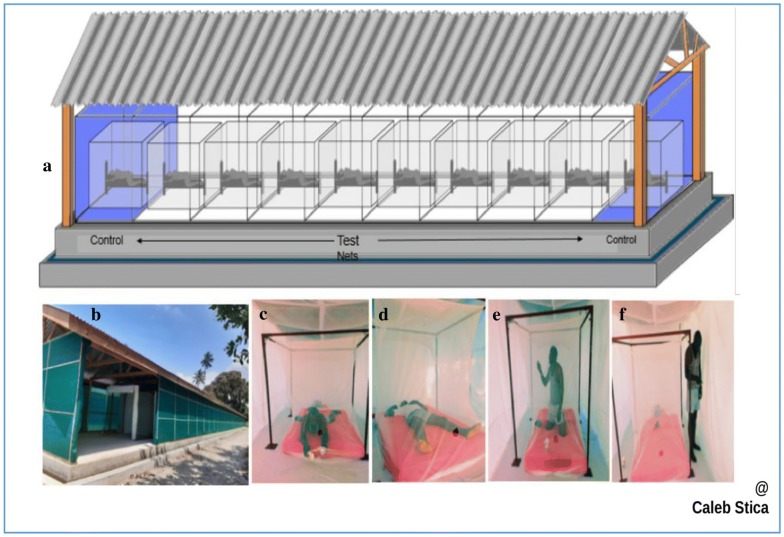
I-ACT assay. **a** Schematic diagram of the Ifakara Ambient Chamber Test (I-ACT with ten chambers); **b** I-ACT at IHI Bagamoyo branch; **c** sleeper releasing mosquitoes within chamber outside the net; **d** volunteer (sleeper) sleeping in side net within a chamber; **e** sleeper collecting mosquitoes using mouth aspirator (siphon) inside net within chamber; **f** sleeper collecting mosquitoes using siphon outside net within chamber

The I-ACT was used as an intermediate between laboratory and experimental hut tests [22]. One LLIN per condition (unwashed or washed 20 times) was tested as a confirmation of the WHO laboratory bioassay findings. Each LLIN or control was randomly assigned to one of the ten testing chambers of the I-ACT (Fig. 2). At 21:00 h, volunteer-sleepers released thirty *An. gambiae* and thirty *An. arabiensis*, in each testing chamber. Mosquitoes were lightly dusted with fluorescent powder (SWADA, Cheshire, United Kingdom) to distinguish the strain as they are morphologically identical. At 06:00 AM, mosquitoes were collected into paper cups using a mouth aspirator. Mosquitoes were scored immediately after collection by strain and sorted into four categories: (1) dead blood-unfed, (2) dead blood-fed, (3) alive blood-unfed and (4) alive blood-fed. Mosquitoes were then held in the testing laboratory at 27 °C ± 5 °C and 40% to 100% relative humidity with access to 10% sugar solution. After 24-h, the proportion of mosquitoes in each of the four categories was again scored using the above criteria. Following each night of the experiment, test nets were re-packed in their respective bags, chambers were cleaned and bed sheets were washed to prevent contamination. LLINs remained fixed to their respective chambers while sleeper volunteers rotated nightly for ten experimental nights so that each volunteer tested each net type once. This was done to account for difference between human attractiveness to mosquitoes that might affect the proportion of mosquitoes blood feeding. Acceptable mortality was ≤ 10% or ≥ 50% blood-feeding success in control [8] (Table 1, Fig. 2).

### Data management and analysis

Data were recorded onto paper forms, double entered into Microsoft excel 2013 and cleaned prior to analysis. Data analysis was performed using STATA 13.1. Descriptive statistics were used for WHO cone and tunnel tests. For the I-ACT, both descriptive statistics and binomial logistic regression with mixed effects were conducted. The outcome measures were 24-h mortality and blood-feeding inhibition. Model fit was tested using AIC [25]. For the model with mortality as the outcome, the best fitting model had treatment and volunteer as fixed effect and day as a random effect while best model with feeding success as the outcome had treatment as a fixed effect, with both volunteer and day as random effects.

## Results

### WHO assays with susceptible *An. gambiae* s.s (Ifakara strain)

#### Olyset^®^

**Fig. 3:**
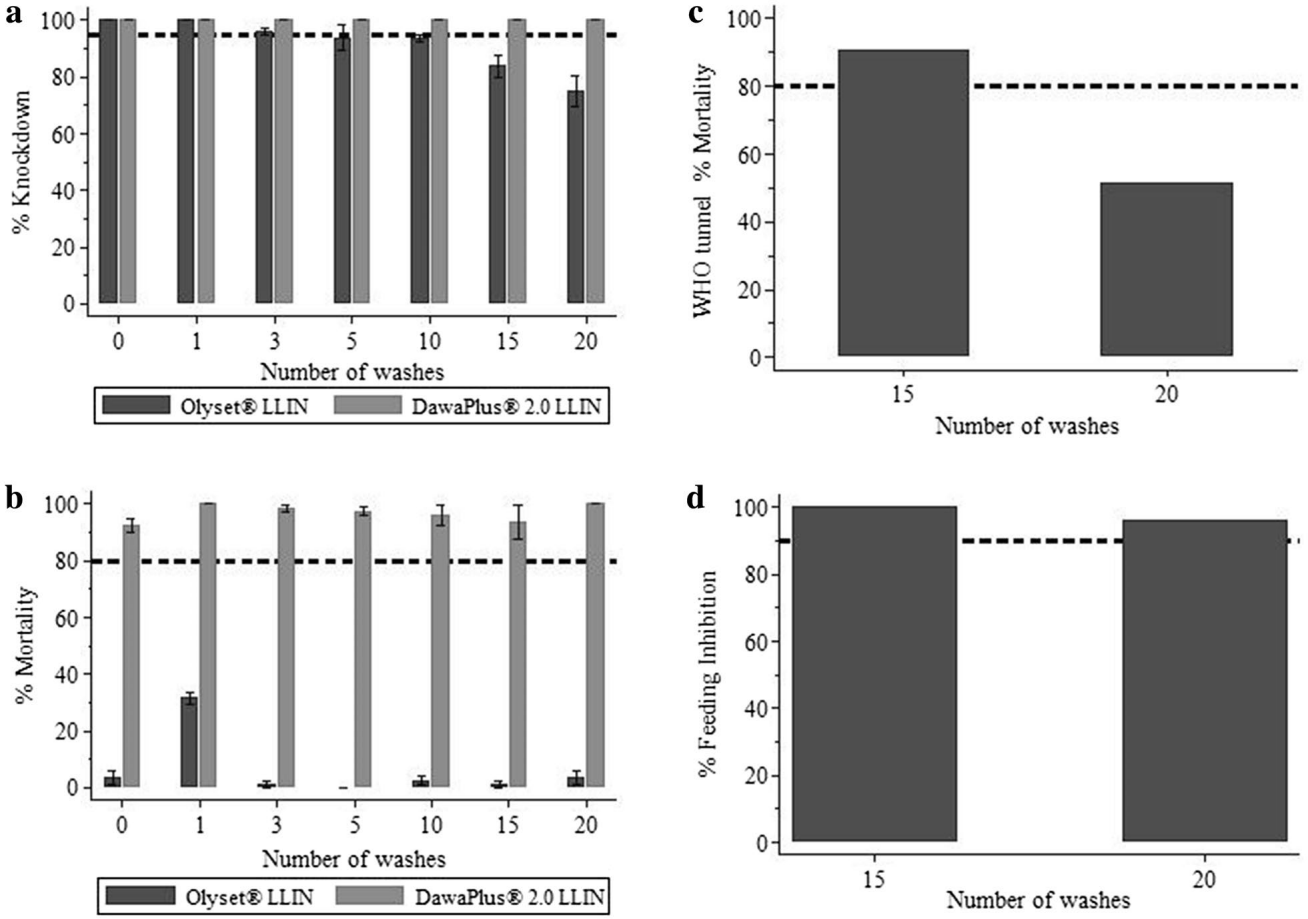
WHO bio-assay results against susceptible *An. gambiae* s.s. (Ifakara strain). **a** Percentage KD60, **b** WHO cone assay percentage 24-h mortality. **c** Tunnel test percentage 24-h mortality, **d** percentage blood-feeding inhibition. In all graphs the dashed line is the WHO cut off criteria, 95% for KD60, 80% for mortality, and 90% for blood-feeding inhibition. **c**, **d** Do not have 95% CI, since only one sample per condition, that failed in cone bioassays was re-tested in WHO Tunnel test as per WHO guideline [8]

LLINs stored for 5 years and 2 months (long storage, LS) fulfilled WHO bio-efficacy criteria up to 20 washes based on the combined WHO cone bioassay and tunnel test against susceptible *An. gambiae* s.s. (Table 2). LS Olyset^®^ LLIN, demonstrated 95% KD60 up to 10 washes in cone bioassay (Fig. 3a) and > 90% feeding inhibition up to 20 washes in tunnel tests (Fig. 3d). Mortality was low in cone bioassays (Fig. 3b).

**Table 2:** WHO bio-assays results against susceptible *An. gambiae* s.s. and resistant *An. arabiensis*

Test system	Test item	Washes	Cone test (N = 80)		WHO tunnel test (N = 100)		Pass/fail WHO efficacy criteria (2013)
			%KD60 [95% CI]	%24-h mortality [95% CI]	%Feeding inhibition [95% CI]	%24-h mortality [95% CI]	
Susceptible *Anopheles gambiae* s.s (Ifakara strain)	Olyset^®^	0	100	03.75 [01.47–06.03]	–	–	Pass
		1	100	31.64 [29.40–33.89]	–	–	Pass
		3	96.25 [95.06–97.44]	01.25 [00.06–02.44]	–	–	Pass
		5	93.75 [89.24–98.26]	0	–	–	Pass
		10	93.75 [92.56–94.94]	02.50 [01.12–03.88]	–	–	Pass
		15	83.75 [79.68–87.82]	01.25 [00.06–02.44]	100	90.91	Pass
		20	75.00 [69.85–80.15]	03.75 [01.47–06.03]	96.00	51.52	Pass
	DawaPlus^®^ 2.0	0	100	92.50 [90.12–94.88]	–	–	Pass
		1	100	100	–	–	Pass
		3	100	98.75 [97.56–99.94]	–	–	Pass
		5	100	97.50 [96.12–98.88]	–	–	Pass
		10	100	96.25 [92.68–99.80]	–	–	Pass
		15	100	93.75 [87.79–99.71]	–	–	Pass
		20	100	100	–	–	Pass
Resistant *Anopheles arabiensis* (Kingani strain)	Olyset^®^	0	38.75 [34.68–42.82]	0	–	–	–
		1	25.00 [19.85–30.15]	01.25 [00.06–2.44]	–	–	–
		3	23.75 [19.68–27.82]	03.75 [01.47–6.03]	96.00	09.18	Pass
		5	51.25 [47.18–55.32]	03.75 [02.56–4.94]	87.00	12.24	Fail
		10	36.25 [33.25–39.25]	01.25 [00.06–2.44]	87.00	14.29	Fail
		15	53.75 [40.79–66.71]	0	92.00	19.39	Pass
		20	58.75 [57.56–59.94]	0	88.00	22.45	Fail
	DawaPlus^®^ 2.0	0	50.00 [44.85–55.15]	10.00 [05.64–14.35]	–	–	–
		1	63.75 [61.47–66.03]	11.25 [06.74–15.76]	–	–	–
		3	67.50 [63.37–71.63]	16.25 [15.06–17.44]	90.00	61.62	Pass
		5	91.25 [87.68–94.82]	36.25 [31.34–41.16]	94.00	56.57	Pass
		10	100	23.75 [19.68–27.82]	94.00	78.79	Pass
		15	97.50 [96.12–98.88]	05.00 [01.63–08.37]	68.00	51.52	Pass
		20	93.75 [89.24–98.26]	12.50 [09.42–15.58]	93.00	41.41	Pass

N= number of mosquitoes released on each test

#### DawaPlus^®^ 2.0

LLINs stored for 4 years and 8 months fulfilled WHO bioefficacy criteria up to 20 washes based on cone bioassay against susceptible *An. gambiae* s.s. (Table 2). LS DawaPlus^®^ 2.0 LLIN, demonstrated 100% KD60 up to 20 washes (Fig. 3a) and > 90% 24-h mortality up to 20 washes (Fig. 3b).

### WHO assays with resistant *Anopheles arabiensis* (Kingani strain)

#### LS Olyset^®^

**Fig. 4:**
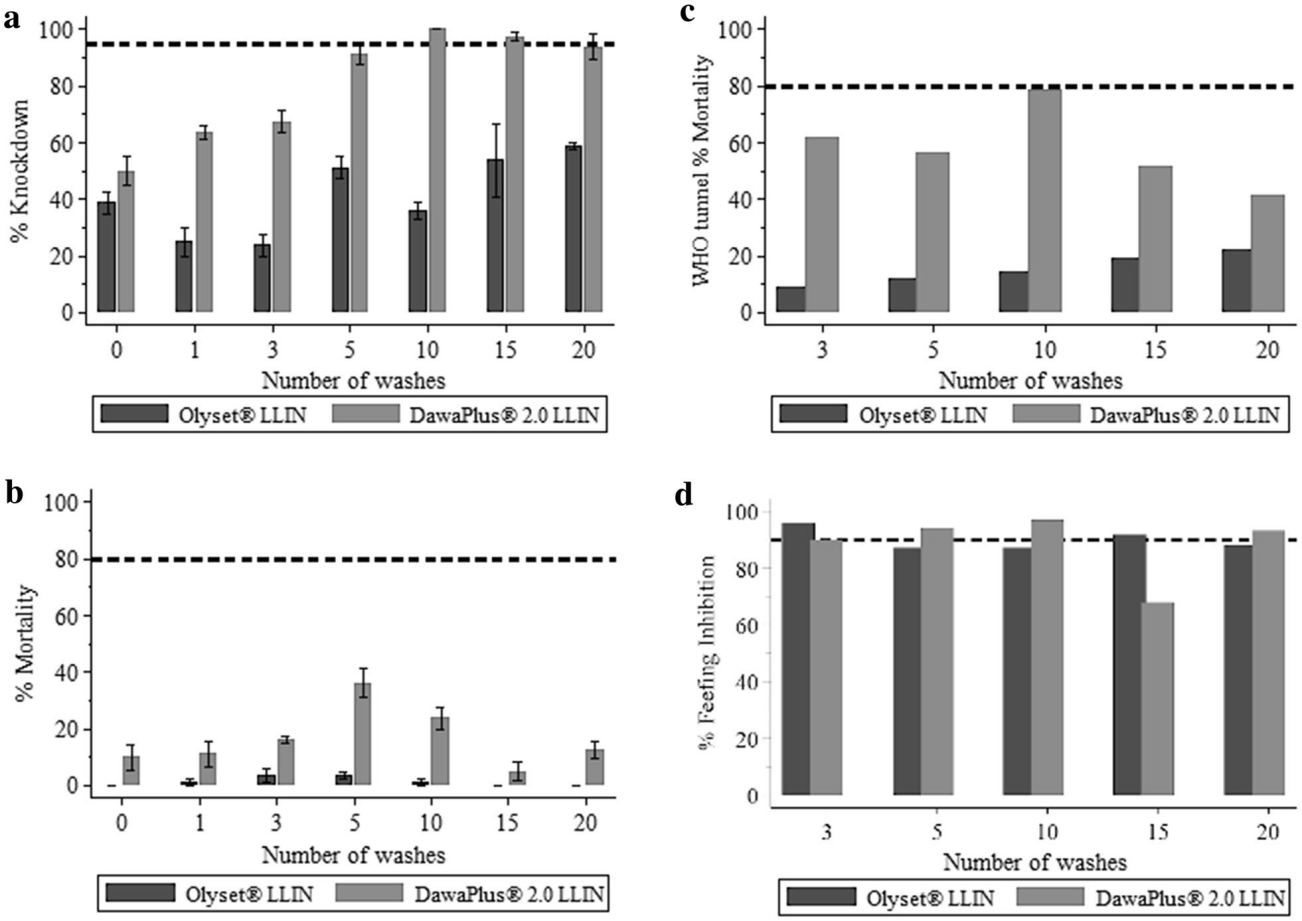
WHO bio-assay results against resistant *An. arabiensis* (Kingani strain). **a** Percentage KD60, **b** WHO cone assay percentage 24-h mortality. **c** Tunnel test percentage 24-h mortality. **d** Percentage blood-feeding inhibition. In all graphs the dashed line is the WHO cut off criteria, 95% for KD60, 80% for mortality, and 90% for blood-feeding inhibition. **c**, **d** Do not have 95% CI, since only one sample per condition that failed in cone bioassays was re-tested in WHO Tunnel test as per WHO guideline [8]

LLINs did not fulfil WHO efficacy criteria up to 20 washes in the combined WHO Cone bioassay and tunnel test against resistant *An. arabiensis* (Table 2). LS Olyset^®^ LLIN did not approach the 95% KD60 threshold in cone tests nor > 80% 24-h mortality (Fig. 4a, c). In tunnel tests, Olyset^®^ LLIN did not approach the 90% feeding inhibition threshold in all tests, except nets washed three and fifteen times demonstrated > 90% feeding inhibition (Fig. 4d). Olyset^®^ did not generate 80% 24-h mortality up to 20 washes (Fig. 4c) in either cone or tunnel tests.

#### LS DawaPlus^®^ 2.0

LLINs fulfilled WHO bioefficacy criteria up to 20 washes based on the combined WHO Cone bioassay and tunnel test against resistant *An. arabiensis* (Table 2). LS DawaPlus^®^ 2.0 LLIN, either demonstrated > 95% KD60 (Fig. 4a) in cone bioassay or > 90% feeding inhibition (Fig. 4d). It did not demonstrate 80% 24-h mortality up to 20 washes (Fig. 4c) in either cone or tunnel tests.

### I-ACT results against susceptible *An. gambiae* s.s. (Ifakara strain)

Against susceptible *An. gambiae* s.s, LS Olyset^®^ and DawaPlus^®^ 2.0 unwashed and washed 20 times exceeded the WHO bio-efficacy criteria for tunnel test on 24-h mortality (≥ 80%) and feeding inhibition (≥ 90%). LS Olyset^®^ and LS DawaPlus^®^ 2.0 nets unwashed and washed 20 times performed similar to new nets of the same brand and washing status, showing almost identical measurements of mortality and feeding inhibition (Table 3). Washing the nets 20 times only marginally reduced their efficacy but still induced high mortality and feeding inhibition, with the old nets nearly as efficacious as the new nets. On the mortality endpoint, LS unwashed Olyset^®^ marginally outperformed the new unwashed Olyset^®^: 99.30% [98.25–100] vs 96.28% [93.64–98.93], odds ratio 0.17 [0.04–0.79] p = 0.024. On the feeding inhibition endpoint, LS DawaPlus^®^ 2.0 washed 20 times marginally outperformed the new Tsara^®^ Soft washed 20 times: 95.62% [92.81–98.42] vs 83.81% [78.98–88.64] OR 4.37 [2.67–7.15], p < 0.0001 (Table 3).

**Table 3:** I-ACT results against susceptible *Anopheles gambiae* s.s and resistant *An. arabiensis*

Test system	Test items	%24-h mortality^a^ [95% CI]	Odds of dying [95% CI]	p-value	% feeding inhibition [95% CI]	Odds of feeding [95% CI]	p-value
Susceptible *Anopheles gambiae* s.s. (Ifakara strain)	LS Olyset^®^ unwashed New Olyset^®^ unwashed	99.30 [98.25–100.0] 96.28 [93.64–98.93]	1.00 0.17 [0.04–0.79]	0.024	94.00 [92.76–99.11] 97.27 [94.84–99.69]	1.00 0.54 [0.05–5.80]	0.610
	LS Olyset^®^ washed New Olyset^®^ washed	85.73 [76.58–94.86] 85.44 [74.29–96.59]	1.00 1.09 [0.61–1.93]	0.775	91.40 [88.66–94.14] 92.29 [88.73–95.86]	1.00 0.84 [0.35–1.99]	0.693
	Old DawaPlus^®^ 2.0 unwashed New DawaPlus^®^ 2.0 unwashed	99.66 [98.88–100.0] 99.65 [98.88–100.0]	–	–	96.12 [94.40–97.83] 89.37 [82.90–95.84]	1.00 2.14 [0.62–7.47]	0.231
	Old DawaPlus^®^ 2.0 washed New DawaPlus^®^ 2.0 washed	100 96.94 [95.56–98.32]	–	–	95.62 [92.81–98.42] 83.81 [78.98–88.64]	1.00 4.37 [2.67–7.15]	0.0001
Resistant *Anopheles arabiensis* (Kingani strain)	Old Olyset^®^ unwashed New Olyset^®^ unwashed	63.40 [47.83–78.97] 50.31 [33.42–67.19]	1.00 0.49 [0.33–0.72]	0.0001	92.10 [88.24–95.95] 95.16 [91.07–99.26]	1.00 0.37 [0.08–1.76]	0.213
	Old Olyset^®^ washed New Olyset^®^ washed	33.34 [17.91–48.77] 37.85 [20.11–55.59]	1.00 1.18 [0.81–1.72]	0.401	84.25 [79.51–88.99] 86.88 [80.34–93.43]	1.00 0.67 [0.29–1.51]	0.329
	Old DawaPlus^®^ 2.0 unwashed New DawaPlus^®^ 2.0 unwashed	71.30 [56.28–86.32] 68.91 [50.64–87.19]	1.00 0.82 [0.53–1.3]	0.364	91.57 [88.72–94.41] 81.78 [75.49–88.07]	1.00 2.55 [1.61–4.06]	0.0001
	Old DawaPlus^®^ 2.0 washed New DawaPlus^®^ 2.0 washed	48.73 [34.18–63.28] 45.74 [27.90–63.57]	1.00 0.86 [0.60–1.22]	0.393	83.28 [76.48–90.08] 59.87 [49.89–69.85]	1.00 4.07 [2.60–6.36]	0.0001

N = 30 mosquitoes released per strain per test

*LS* long storage

^a^Arithmetic mean control-corrected 24-h mortality with 95% confidence intervals (CI) and Arithmetic mean blood-feeding inhibition with 95% confidence intervals (CI)

### I-ACT results against resistant *An. arabiensis* (Kingani)

Against resistant *An. arabiensis*, unwashed LS Olyset^®^ and unwashed LS DawaPlus^®^ 2.0 exceeded the WHO bio-efficacy criteria for tunnel tests on feeding inhibition (≥ 90%). All the net types and condition failed to meet WHO bioefficacy criteria on 24-h mortality (≥ 80%) against the resistant strain. Olyset^®^ and DawaPlus^®^ 2.0 LS nets unwashed and washed 20 times performed in a similar way to new nets of the same brand and washing status on both endpoints showing almost identical mortality and feeding inhibition (Table 3). As was observed with the susceptible strain, on the mortality endpoint, LS unwashed Olyset^®^ marginally outperformed the new unwashed Olyset^®^ 63.40% [47.83–78.97] vs 50.31% [33.42–67.19], odds ratio 0.49 [95% CI 0.33–0.72], p < 0.0001 (Table 3). On the feeding inhibition endpoint, LS unwashed DawaPlus^®^ 2.0 outperformed the new unwashed DawaPlus^®^: 91.57% [88.72–94.41] vs 81.78% [75.49–88.07], OR 2.55 [1.61–4.06], p < 0.0001 (Table 3). Additionally, on the feeding inhibition endpoint, LS DawaPlus^®^ 2.0 washed 20 times outperformed the new DawaPlus^®^ 2.0 washed 20 times: 83.28% [76.48–90.08] vs 59.87% [49.89–69.85], OR 4.07 [2.60–6.36], p < 0.0001 (Table 3).

## Discussion

This study provides valuable information on the effect of long storage conditions on the bio-efficacy of LLINs for malaria control programmes. The study showed that LLINs remained efficacious despite being stored for about 5 years under controlled storage conditions. The nets used for this study were pyrethroid of two types: Olyset^®^, a permethrin incorporated net, and DawaPlus^®^ 2.0 (Tsara^®^ soft), a deltamethrin coated net with insecticide held to the filaments using a binder.

It was necessary to keep the investigational LLINs under ideal temperature and humidity conditions, as it is known that high temperature may inactivate the insecticide or binder [19, 26]. Proper storage should also avoid direct sunlight as pyrethroids are decomposed by UV light and heat [27]. Several studies have been conducted to evaluate the storage conditions of LLINs. For example, the study conducted in Turkey by Karakus et al. reported that nets exposed to direct sunlight for 6 months, had lower efficacy (44.4% 24-h mortality), than other groups of nets which were not exposed to sunlight (100% 24-h mortality) [19]. Atieli et al. showed that drying methods used after washing nets, resulted in significant impact on the efficacy of pyrethroid nets: nets washed 20 times and dried under the shade retained more pyrethroid insecticide (62.5%) than nets directly dried under the sunlight (58.8%) [20]. Furthermore, Peck et al. reported that the insecticidal activity of the pyrethroid Lambda-cyhalothrin was reduced after 10 weeks of exposure to direct sunlight [26].

LLINs are designed to withstand the high temperatures that may be encountered in the tropics and the findings from this study suggest that nets can retain bio-efficacy for up to 5 years if stored out of sunlight at the range of 25 °C to 33.4 °C and 40% to 100% relative humidity. The storage conditions used in this study aligned with the manufacturer specification and WHO guidelines [27, 28]. Therefore, national malaria control programmes (NMCPs) and other stakeholders should be well informed on the appropriate long-term storage conditions for pyrethroid nets in order for the LLINs to retain their bio-efficacy, if nets are to be stored for extended period before distribution. NMCPs also advised to invest on storage facilities that use shading and passive heat transfer similar to that of the Ifakara Health Institute (Fig. 1) at a low running cost to ensure efficacy of LLINs after storage.

The performance of long storage (LS) LLINs varied between net brands and washes in the WHO cone bioassay. DawaPlus^®^ 2.0 LLIN, met the WHO criteria in the standard WHO cone assay without the need to conduct a WHO tunnel test, while Olyset^®^ LLIN failed to meet the criteria based on the cone assay but passed based on WHO tunnel test (Table 2). This is because Olyset^®^ is a high density polyethylene net, meaning that migration of permethrin is very slow with short wash intervals, hence surface concentrations are very low, sufficient to induce KD60 effect, but insufficient to induce mortality [22, 29, 30]. This mode of action reduces the probability of mosquito dying from exposure to the insecticide following multiple contacts with net, but also gives Olyset^®^ its feeding inhibition properties that were observed in the I-ACT (Table 3), allowing protection of human volunteers sleeping beneath them even after 5 years and 2 months of storage. Similar results were observed by Massue et al. [22]. It was again observed, by Jaramillo et al., on which permethrin treated net (Olyset^®^ LLIN) reduced contacts of *Anopheles albimanus* to net surface in the cone test [31].

Both LS Olyset^®^ and DawaPlus^®^ 2.0 performed well on the feeding inhibition end point against the resistant *An. arabiensis* (Kingani strain), this is crucial because the results suggest that pyrethroid nets may still confer blood feeding protection against resistant mosquitoes due to irritancy [32]. However, it is clear that LLIN performance was not significantly impaired as a result of long storage, but due to ability of the resistant strain to detoxify pyrethroids [33–35]. It is for this reason that piperonyl butoxide-treated insecticidal nets (PBO) nets have been developed [36]. PBO is a synergist commonly used in pest control, combined with pyrethroid, that hinders enzymatic detoxification of pyrethroids that enables the survival of individuals with upregulated detoxification phenotypes, and allow the pyrethroids insecticide to finally kills pyrethroid resistant mosquitoes [37]. It is planned to conduct further studies to investigate the long-term storage stability of nets treated with PBO in the future using the set up described here. Although, it is interesting that both nets still performed well on the feeding inhibition end point, which means that long stored pyrethroids LLINs can still confer protection, therefore, reiterate the usefulness in the continuous control of mosquitoes.

Results from the I-ACT with volunteers sleeping beneath the LLINs complemented the evidence provided by the WHO cone assays and allowed for comparison between new nets and long storage nets of the same brand and washing status. Using WHO pass/fail thresholds, findings from WHO cone assays and the I-ACT with LS nets agreed between net brands and washes. Although, using the WHO criteria, both LS nets and new nets passed with the susceptible strain but gave inconsistent results with the resistant strain. The I-ACT demonstrated higher feeding inhibition and mortality (Tables 2 and 3). The increased performance of LLINs in the I-ACT might be due to extended exposure time that increased number of contacts between mosquitoes and the LLIN, use of a whole net and the use of a preferred (human) bait by mosquito and larger surface are of net presented to the mosquitoes. Similar I-ACT results have also been observed by Massue et al. [22]. However, findings from current study shown that, long storage nets performed similarly to the new nets in the I-ACT on both mortality and feeding inhibition endpoints.

### Study limitations

The study was conducted as per protocol and WHO guidelines for LLIN evaluations. However, the I-ACT study was not sufficiently powered (< 80%) and one net per condition was used, which limited the study to adequately measure inter-net heterogeneity due to limited number of test nets available in the facility. The bursting strength of nets and chemical analysis was not evaluated and this needs to be considered to understand the effect of storage on the fabric strength and amount of insecticides present. Therefore, the findings of the study should be cautiously interpreted and we recommend further studies to be conducted in multiple sites with sufficient power to detect differences between nets for each condition, and additional evaluations of bursting strength after storage.

## Conclusion

Even after long-term storage of around 5 years, Olyset^®^ and DawaPlus^®^ 2.0 (now called Tsara^®^ soft) LLINs remain efficacious against susceptible *Anopheles* mosquitoes at optimal storage range of 25 °C to 33.4 °C for temperature and 40% to 100% relative humidity measured by standard WHO methods. DawaPlus^®^ 2.0 (Tsara^®^ soft) also, passed WHO efficacy criteria on unwashed LLINs and after 20 washes against resistant *An. arabiensis.* These data were confirmed in the I-ACT. Therefore, long stored nets can still be useful in controlling malaria in endemic areas when optimal storage conditions of nets is maintained.

## Data Availability

All data are available at Ifakara Health Institute archive.

## Abbreviations

ACT: Artemisinin-based combination therapy
AIC: Akaike information criterion
I-ACT: Ifakara ambient chamber test
IHI: Ifakara Health Institute
KD60: Knockdown at 60 min
LLINs: Long-lasting insecticidal nets
LS: Long storage
NGOs: Non-governmental organizations
OR: Odds ratio
PBO: Piperonyl butoxide
PMI: President’s malaria initiative
RH: Relative humidity
VCPTU: Vector control product testing unit
WHO: World Health Organization
